# Supplementary material for: The Impact of Processing Parameters on the Content of Phenolic Compounds in New Gluten-Free Precooked Buckwheat Pasta
Source: Molecules. 2019 Apr 1;24(7):1262. doi: 10.3390/molecules24071262 (PMC6480078; doi:10.3390/molecules24071262)
Supplement: Supplementary file 1 [file molecules-24-01262-s001.pdf]

supplementary materials

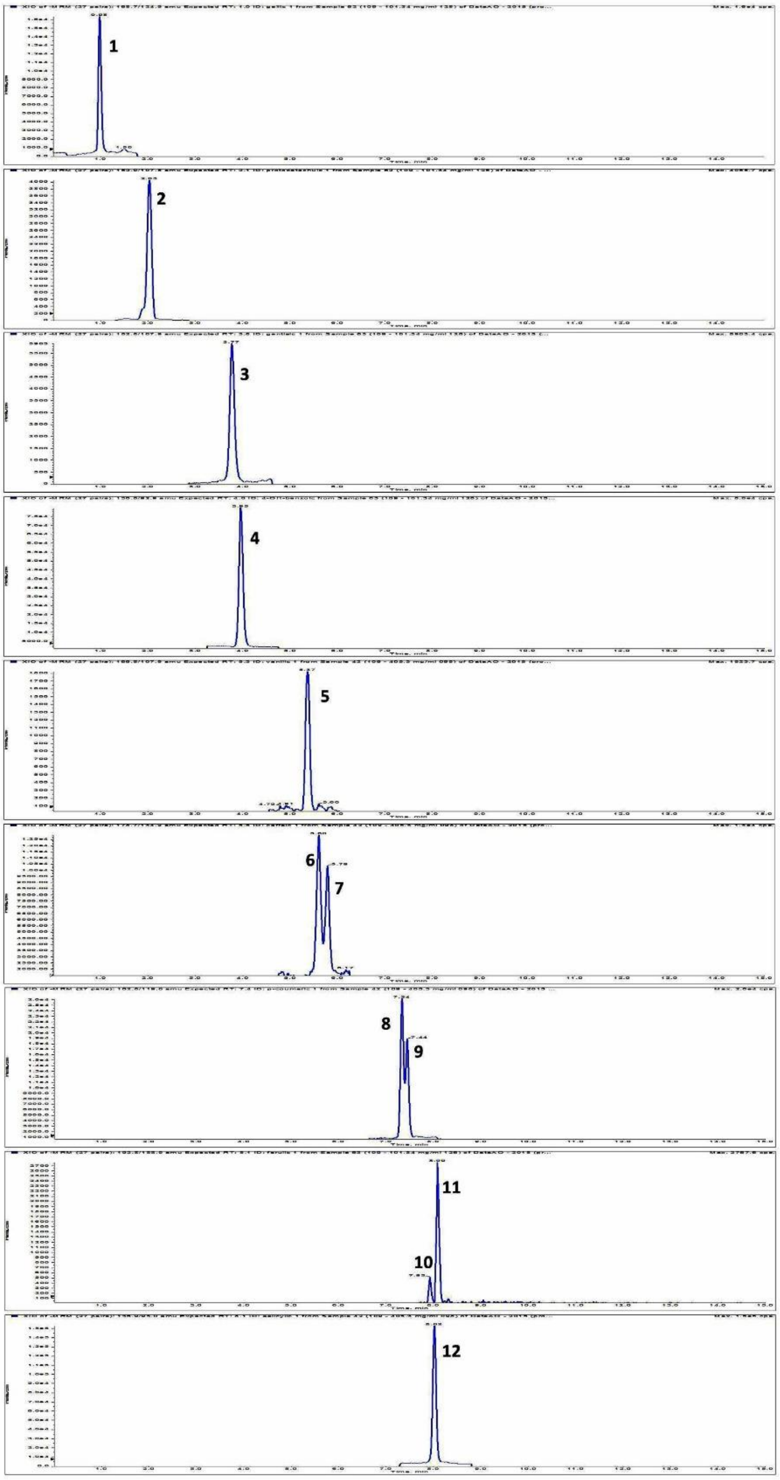

Figure S1. Exemplary LC-MS/MS chromatogram of phenolic acids from precooked buckwheat pasta**.** **1**. gallic acid, **2.** protocatechuic acid, **3.** gentisic acid, **4.** 4-OH-benzoic acid, **5.** vanilic acid, **6.** *trans*-caffeic acid, **7.** *cis*-caffeic acid, **8**. *trans*-p-coumaric acid, **9**. *cis*-p-coumaric acid, **10.** *trans*-ferulic acid, **11.** *cis*-ferulic acid, **12.** salicylic acid
